# [Corrigendum] Androgen deprivation therapy has no effect on Pim-1 expression in a mouse model of prostate cancer

**DOI:** 10.3892/ol.2026.15911

**Published:** 2026-10-19

**Authors:** Jiang Wang, Gang Li, Bo Li, Hualin Song, Zhiqun Shang, Ning Jiang, Yuanjie Niu

Oncol Lett 13: 4364-4370, 2017; DOI: 10.3892/ol.2017.6010

Following the publication of the above article, an interested reader drew to the authors' attention that, regarding the immunohistochemical experiments shown in Fig. 6 on p. 4367, the “ADPC SABC x200” and “ADT SABC x200” data panels (Fig. 6A and B respectively) showed an overlapping section, such that data which were intended to show the results of differently performed experiments had apparently been derived from the same original source.

The authors were able to consult their original data, and realize that the panel selected for Fig. 6B had inadvertently been chosen incorrectly. A revised version of Fig. 6, now showing the correct data for the “ADT SABC x200” panel, is shown below. The authors regret that this error went uncorrected in the originally published version of this figure, although this did not grossly affect either the results or the conclusions reported in this article. All the authors agree with the publication of this Corrigendum, and thank the Editor of *Oncology Letters* for granting them the opportunity to publish this; furthermore, they apologize to the readership for any inconvenience caused.

## Figures and Tables

**Figure 6.**
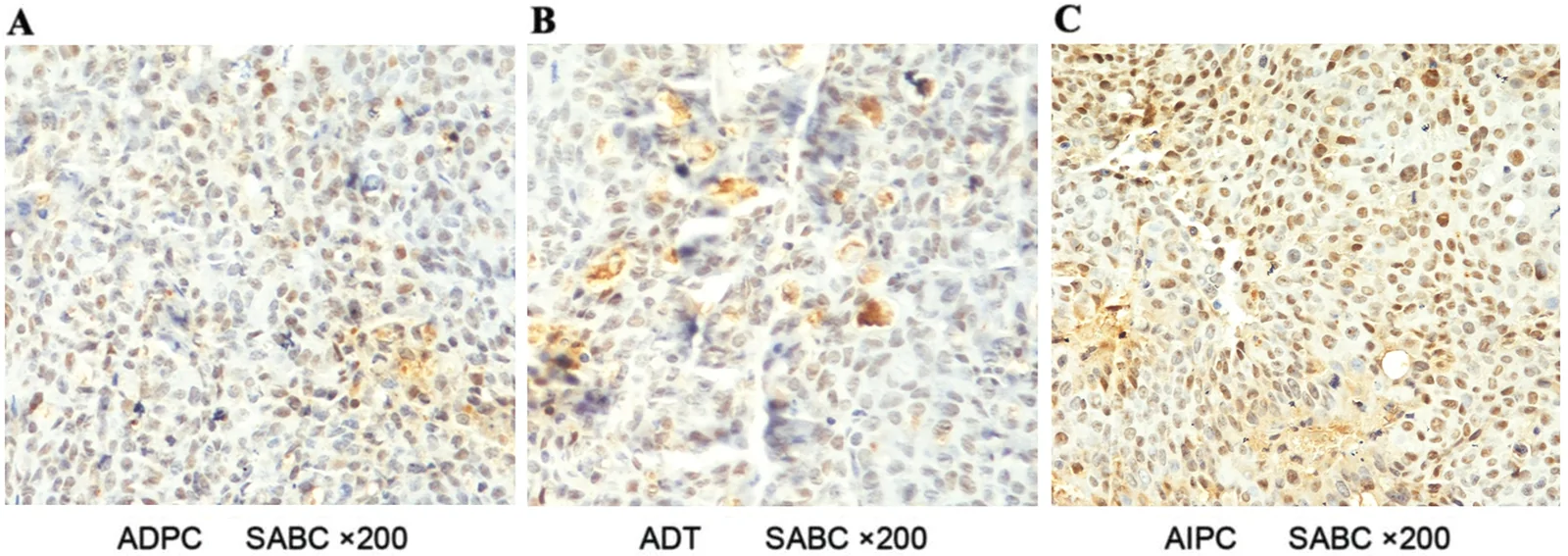
Immunohistochemical staining revealed Pim-1 expression was increased from ADPC to AIPC in orthotopic prostatic implantation tumors (magnification, ×200). (A) Pim-1 was positive in the cytoplasm of tumor cells in the ADPC group. (B) Positive Pim-1 expression was increased following ADT. (C) The majority of the cancer cell cytoplasms were strongly stained for Pim-1 in the AIPC group. ADPC, androgen-dependent prostate cancer; AIPC, androgen independent prostate cancer; ADT, androgen deprivation therapy; SABC, streptavidin-biotin complex.

